# Shared health governance, mutual collective accountability, and transparency in COVAX: A qualitative study triangulating data from document sampling and key informant interviews

**DOI:** 10.7189/jogh.13.04165

**Published:** 2023-12-08

**Authors:** Ariel Gorodensky, Quinn Grundy, Navindra Persaud, Jillian C Kohler

**Affiliations:** 1 Leslie Dan Faculty of Pharmacy, University of Toronto, Toronto, Ontario, Canada; 2 Lawrence Bloomberg Faculty of Nursing, University of Toronto, Toronto, Ontario, Canada; 3 Dalla Lana School of Public Health, University of Toronto, Toronto, Ontario, Canada; 4 Munk School of Global Affairs, University of Toronto, Toronto, Ontario, Canada

## Abstract

**Background:**

To facilitate global COVID-19 vaccine equity, the World Health Organization, the Coalition for Epidemic Preparedness Innovations, the Global Alliance for Vaccines and Immunizations, and the United Nations Children’s Fund supported the COVID-19 Vaccine Global Access (COVAX) partnership. COVAX’s goals may have best been pursued through shared health governance – a theory of global health governance based on six premises, in which global health actors collaborate to achieve a shared goal. Shared health governance employs a framework for accountability termed “mutual collective accountability”, in which actors hold each other accountable for achieving their goal, thus relying on transparency with one another.

**Methods:**

We conducted a multi-method qualitative study triangulating document analysis and key informant interviews to address the question: To what extent did COVAX employ shared health governance, mutual collective accountability, and transparency? We thus aimed to explore the governance structures and accountability and transparency mechanisms in COVAX and determine whether these constituted shared health governance and mutual collective accountability.

**Results:**

We identified 117 documents and interviewed 20 key informants. Our findings suggest that COVAX’s co-convening organisations were governed by their individual formal governance mechanisms, while each was formally accountable to its own leadership team, resulting in challenges when activities and decisions involved collaboration between organisations. Furthermore, COVAX’s governance lacked transparency, as there was little public information about their decision-making processes and operations, including information about the algorithm with which they make vaccine allocation decision, possibly contributing to its inability to achieve its goals.

**Conclusions:**

The COVAX partnership only achieved four of the six premises of shared health governance. Since actors involved in COVAX did not hold one another accountable for their role in the partnership, it did not employ mutual collective accountability, while also lacking in transparency. Although these results do not entirely explain COVAX’s shortcomings, they contribute to evidence about the roles of good governance, transparency, and accountability in large global health initiatives and underscore failures of the current global governance system.

The World Health Organization (WHO) declared COVID-19 a public health emergency of international concern on 30 January 2020 and a pandemic on 11 March 2020 [1]. The emergence of variants such as Omicron due to mutations exposed weaknesses in health systems around the world [2,3], while their rapid spread across national borders indeed highlighted how health systems are *“*mutually interdependent and share vulnerabilities” [4]. To address these vulnerabilities during the pandemic, governments and international institutions had to urgently collaborate to promote non-discriminatory and global equitable access to COVID-related health care [5].

Specifically, mitigating the spread of disease and the burden of the pandemic required global, equitable distribution and administration of COVID-19 vaccines [6], crucial in reducing mortality and morbidity [7]. Vaccine distribution, however, was guided by vaccine nationalism, which prioritised national public health and domestic economies over health equity, thus failing to address the interdependencies and mutual vulnerabilities that threaten global health [4,8,9].

To tackle these global inequities in access to COVID-19 vaccines, the WHO, the Coalition for Epidemic Preparedness Innovations (CEPI), the Global Alliance for Vaccines and Immunizations (Gavi), and the United Nations Children’s Fund (UNICEF) (termed co-convening organisations) set up the COVID-19 Vaccine Global Access (COVAX) partnership [10] in April of 2020 as part of the Access to COVID-19 Tools (ACT) Accelerator, a multilateral effort to facilitate the global equitable distribution of COVID-19 tools [11]. The ACT Accelerator had four pillars, each supporting distribution of one type of tool: COVID-19 therapeutics, diagnostics, vaccines, or general emergency health system support [12].

COVAX, the ACT Accelerator’s vaccine pillar, was established with the purpose of ending the acute phase of the pandemic by the end of 2021 by facilitating the global equitable distribution of COVID-19 vaccines [13]. This was to be achieved by first “…pool(ing) resources and shar(ing) vaccine development risk” to diversify vaccine portfolios, then by facilitating the deployment of safe, effective, and affordable COVID-19 vaccines worldwide [14].

The procurement and deployment of COVID-19 vaccines was done through the partnership’s COVAX Facility, which was the global pooled mechanism for procuring COVID-19 vaccines [15]. COVAX’s initial goal was to vaccinate 20% of all participating countries’ populations by the end of 2021, which would require the procurement and delivery of an estimated two billion vaccine doses via the COVAX Facility [13].

While COVAX had the potential to contribute to global equitable vaccine deployment, it did not achieve its initial goal, delivering 910.4 million vaccine doses in 2021 [16]. In September 2021, COVAX expanded its operations to become a global mechanism for COVID-19 vaccine sharing [17]. In 2021, 60% (543 million of the total 910 million) of the COVID-19 vaccine doses delivered via the COVAX Facility were provided through this mechanism [17]. As of September 2023, COVAX still had not reached its two billion dose goal [16]. The volume of its donations never became sufficient to achieve global vaccine equity.

## Shared health governance and mutual collective accountability

There was a global public interest in ensuring that COVAX ended the acute phase of the COVID-19 pandemic by the end of 2021. Prah Ruger argues that when “(w)e all have an interest in health protection and promotion”, as is the case in COVAX and with the distribution of COVID-19 vaccines, serving these interests requires good global health governance [18]. She proposes and has applied a specific framework for global health governance that is appropriate when a global health initiative involves shared responsibility, resources, and action, naming it “shared health governance” [4,19-23]. This framework “…calls for developing a shared vision of health and health provision by amalgamating values among global, national, and local actors” [4,19,21] by employing social rationality, which “…recognizes that health is a collective interest of both self and other” [4,19], thus prioritising global health over national interests [4]. Social rationality is of particular importance for COVAX, as the partnership aims to promote global COVID-19 vaccine equity, but does not intend to disregard the individual interests of its participating nations [24].

Prah Ruger explains that shared health governance has six basic premises (**Table 1**) [22]. Operationalising these requires that global health actors hold each other accountable for their role in a given initiative. Specifically, shared health governance employs a specific framework for accountability termed “mutual collective accountability” [4], which is functionally a “…checks-and-balances system” that fosters mutual regulation [19,21]. Under it, global health actors hold each other to account for their contribution to the agreed-upon global health outcome(s): in the case of COVAX, the global equitable distribution of COVID-19 vaccines.

**Table 1:** Prah Ruger’s six premises of shared health governance

Shared health governance [22]	
**Premise number**	**Description**
1	A variety of social actors must collaborate to produce the conditions for everyone to be healthy
2	Every actor involved in an initiative must commit to producing the conditions for everyone to be healthy
3	Actors must commit to a shared ideal or set of ideals through which they achieve a shared purpose
4	All actors must share resources fairly
5	Actors must agree upon a system of social sanctions to be imposed if anyone deviates from the shared goals of the initiative
6	Actors must share sovereignty and constitutional commitment for the shared initiative

## Transparency

Transparency is a necessary, though not a sufficient, condition for accountability [25]. This means that, for institutions or organisations to hold one another accountable for achieving a shared global health goal, they must be transparent with one another. Consequently, without transparency, mutual collective accountability, and in turn, shared health governance, cannot be achieved [8]. Transparency can be defined in many ways. Here we adopt the definition offered by Transparency International, describing it as “…all about knowing who, why, what, how and how much. It means shedding light on formal and informal rules, plans, processes and actions” [26].

Specifically, transparency in the global distribution of COVID-19 vaccines and within COVAX should include clinical trial data transparency, the publication of complete and un-redacted COVID-19 vaccine procurement contracts, vaccine pricing transparency, and the publication of vaccine delivery schedules [27]. This would provide vaccine-recipient governments and other actors in COVAX with the information necessary to hold vaccine manufacturers accountable for the timely and equitable deployment of safe, effective, and affordable vaccines.

## Rationale

Existing literature highlights the need for good governance, which includes concepts such as transparency and accountability, in the distribution of COVID-19-related products [28-33], and also outlines the importance of global vaccine equity in the COVID context and the potential lessons to be learned from COVAX [34-36]. Additionally, some literature [4,8,37] has stressed the importance of employing shared health governance and mutual collective accountability in the global distribution of emergency health products during COVID-19; there is no literature, however, about the use or application of these frameworks in COVAX specifically. This leaves an important research gap to be filled, since many countries in 2021 relied solely on COVAX for access to COVID-19 vaccines [38], and theoretical perspectives grounded in health equity and justice suggest that good governance, accountability, and transparency mechanisms could have furthered the initiative’s goal [4,19-22]. Thus, we sought to analyse the COVAX Facility structures and activities to understand to what extent it employed shared health governance, mutual collective accountability, and transparency. Ultimately, it is crucial to understand the global health governance, transparency, and accountability mechanisms that could have successfully facilitated global equitable vaccine distribution. We aimed to explore the types of governance structures and accountability and transparency mechanisms that exist within COVAX and determine whether these constitute shared health governance and mutual collective accountability.

## METHODS

We conducted a multi-method qualitative study design involving document sampling and key informant interviews, which we grounded in a critical realist perspective [39,40]. By triangulating data sources, we sought to generate a comprehensive description and analysis of governance, transparency, and accountability mechanisms in COVAX. We reported our study according to Consolidated Criteria for Reporting Qualitative Research (COREQ) guidelines [41] (File S1 in the **Online Supplementary Document**). We received ethical approval from the University of Toronto Research Ethics Board (#00041980).

### Document sampling

Document sampling involved iteratively and purposively searching for electronic documents/webpages that described at least one aspect of governance, transparency, and/or accountability in COVAX. Between November 2021 and January 2022, AG manually searched COVAX websites and websites of its co-convening organisations and websites of other international organisations with connections to COVAX or global COVID-19 vaccine distributions (e.g. Transparency International, U4 Anti-Corruption Resource Centre). Due to the lack of published literature on this topic, we did not search through peer-reviewed academic literature.

### Key informant interviews

We recruited interview participants via email using purposive and snowball sampling [42,43], beginning with personal contacts and individuals found on institutional websites. We conducted key informant interviews with individuals who worked for a COVAX co-convening organisation or who were otherwise involved in the partnership, those who worked for non-governmental organisations (NGOs) involved in work related to/or who conducted research about COVID-19 vaccine distributions, or journalists who had reported/were reporting on COVAX’s role in the global distribution of COVID-19 vaccines. We interviewed these groups because they had credible, first- or second-hand information about COVAX’s operations.

AG conducted all interviews between February and March of 2022 via Zoom; all interviews were semi-structured and conducted in English (File S2 in the **Online Supplementary Document**). Participants provided written consent and were given the option to have their interviews audio recorded or to have notes taken about the information they provided.

### Data extraction and analysis

We took a deductive qualitative content analysis approach to data analysis [44-46] by first creating a deductive coding framework (File S3 in the **Online Supplementary Document**) based on the six premises of shared health governance and the framework of mutual collective accountability. We wrote a memo for each code containing a descriptive summary and an interpretation of the data under it, and we used these memos to describe our findings.

## RESULTS

We identified 499 publicly-available documents/webpages and, after removing documents without information about governance, transparency, and/or accountability in COVAX and duplicates, we included 117 in our analysis (**Table 2** and File S4 in the **Online Supplementary Document**).

**Table 2:** Breakdown of documents/webpages

Source	Number of documents/webpages included
Gavi	22
CEPI	8
WHO	18
UNICEF	23
COVAX partnership (joint publication involving all COVAX convening organisations)	28
Other	18
**Total**	117

We invited 63 individuals to participate in key informant interviews and 20 agreed to participate. Eight were employees of COVAX co-convening organisations (or individuals otherwise involved in COVAX), nine were NGO employees, and three were journalists. Twenty-one individuals did not respond to recruitment emails and 22 individuals stated that they could not participate. Among the reasons for declining an interview request was lack of time, not being at liberty and/or in the position to discuss governance, transparency, and/or accountability in COVAX, or the presence of confidentiality/non-disclosure agreements with their employers that precluded them from speaking with us.

Interview participants represented individuals from each of COVAX’s co-convening organisations, three media outlets, and seven NGOs. Nine participants were located in North America (45%), eight in Europe (40%), two in Africa (10%), and one in South America (5%). Interviews were between 20 and 45 minutes in duration (mean = 31 minutes) and all were individual expect for one which involved two participants from the same organisation.

We stopped recruitment four interviews after we had reached meaning saturation [47], which occurred when further interviews of individuals of the same group (either internal or external to COVAX) no longer provided new information; this occurred after five interviews with informants involved in the partnership and 12 interviews with informants external to COVAX. After saturation had been reached for each group, we interviewed one more individual external to COVAX and three more individuals from within co-convening organisations.

### Governance

To ensure that COVAX was established and could begin its operations rapidly, its co-convening organisations agreed not to create new formal institutions or governance structures. Instead, each co-convening organisation was governed by its own independent board. Specifically, each co-convening organisation carried out a specific role tailored to their expertise, the operation of which was overseen by their individual boards. In this sense, CEPI oversaw vaccine research and development (R&D) and scaling up of the production of COVID-19 vaccines [48-50]. Gavi oversaw the Gavi COVAX Advanced Market Commitment, the financing mechanism established to support the participation of the Facility’s Advanced Market Commitment participants [48,51], and was also the legal administrator of the COVAX Facility [15]. The WHO was involved in COVAX in normative and regulatory roles [48]. For example, they established the COVAX Facility’s Fair Allocation Framework, a guidance document for the distribution of vaccines between countries, and provided policy recommendations for vaccine regulation and advice on the emergency licensure of vaccines [48,52]. Lastly, UNICEF was the procurement and in-country partner for COVAX [48]. They work collaboratively with COVID-19 vaccine manufacturers, freight operators, airlines, and logistics and storage experts to ensure that vaccine-recipient countries receive the vaccines and ancillary items (i.e. syringes) necessary for successful vaccine rollouts [53,54].

An interview participant from a co-convening organisation explained that, when COVAX was created, “*one of the founding principles was not to create new institutions and instead to rely upon existing institutions to the extent possible. So each of the respective institutions maintain[ed] their own formal governance processes*” (COVAX participant 7).

According to an interviewee, all other governance mechanisms within COVAX were considered *“informal”* (COVAX participant 7), meaning that they occurred outside of any single organisation’s established structures. Specifically, COVAX’s operations were divided into three workstreams: The Development and Manufacturing, Procurement and Delivery at Scale, and Policy and Allocation workstreams, which supported vaccine manufacturing and development, procurement and delivery, and allocation, respectively [55]. Each of these workstreams had informal governance bodies that involved a few COVAX actors (**Table 3**).

**Table 3:** Description of COVAX’s workstreams and their governance bodies

	Development and manufacturing workstream	Procurement and delivery at scale workstream	Policy and allocation workstream
**Descriptio**n	This workstream manages the development and manufacturing of COVID-19 vaccines [55].	This workstream manages the procurement of COVID-19 vaccines. It involves the COVAX Facility [55].	This workstream provides policy recommendations and manages the allocation of vaccines procured via the COVAX Facility [55].
**Governance bodies**	Research and Development and Manufacturing Investment Committee [55]: Makes investment recommendations for the COVAX Facility's vaccine portfolio. Involves Richard Hatchett (CEPI CEO), Seth Berkley (Gavi CEO), Bill and Melinda Gates Foundation President of Global Health, and representatives from the pharmaceutical industry.	Gavi Board [15,56-58]: 28 members from a variety of backgrounds. Holds ultimate responsibility for the COVAX Facility. Hosts the Office of the COVAX Facility, through which it governs and supports the Facility's operations.	WHO Strategic Advisory Group of Experts on Immunization Working Group on COVID-19 Vaccines [55,57-61]: Advises WHO Member States on the use of and licensure of COVID-19 vaccines.
	Technical Review Group [55]: Provides technical guidance, review, and oversight for projects supported by the Research and Development and Manufacturing Investment Committee. Has involvement from the WHO, Gavi, CEPI, and other global health organisations.	COVAX Shareholders Council [15,57,62-65]. Self-organising group of self-financing participants. Provides guidance and advice to the Office of the COVAX Facility.	Joint Allocation Taskforce [15,5155,86-89]: Makes vaccine allocation decisions. Passes these decisions onto the independent allocation of vaccines group for review. Involves individuals from the Office of the COVAX Facility and the WHO.
	SWAT teams [50,55]: Focused on resolving technical issues and challenges related to vaccine development. There are 3 SWAT teams: Enabling Sciences team, Manufacturing team, Clinical development team	Advanced Market Commitment Engagement Group [15,58,64-67]: Self-organising group of Advanced Market Commitment participants. Provides guidance and advice to the Office of the COVAX Facility.	Independent Allocation of Vaccines Group [15,51,55,56,61,68-70]: 12 technical experts. Validates the Joint Allocation Taskforce's vaccine allocation decisions. Ensure that these decisions are transparent, devoid of conflict of interest, and technically-informed. Pass allocation decisions onto the Office of the COVAX Facility for implementation
	Regulatory Advisory Group [50,55]: Provides guidance to each of the SWAT teams.	COVAX Consensus Group [15,55-57]: Supports decision-making in the COVAX Facility. Composed of the Chair and Vice Chair of the Gavi Board, the Shareholders Council, the Advanced Market Commitment Engagement Group, and the heads of the co-convening organisations.	
		Independent Product Group [15,55,57,67,71,72]: 10 members from a variety of academic and global health institutions. An advisory board that makes recommendations for the inclusion/exclusion of vaccines in the Facility, gives overall guidance on the Facility's vaccine portfolio.	
		Procurement Reference Group [15,55,71]: 5-7 members with expertise in vaccine manufacturing, procurement, and delivery. An advisory board that makes recommendations for the Facility's vaccine portfolio and on how the Facility should manage financial risks.	
		Gavi Market-Sensitive Decisions Committee [55,57,73]: 15 members from a variety of global health and civil society organisations. Reviews agreements between the COVAX Facility and vaccine manufacturers.	

COVAX – COVID-19 Vaccine Global Access, Gavi – Global Alliance for Vaccines and Immunization, WHO – World Health Organization, CEPI – Coalition for Epidemic Preparedness Innovations, SWAT – Support Work to Advance Team

**Figure 1:**
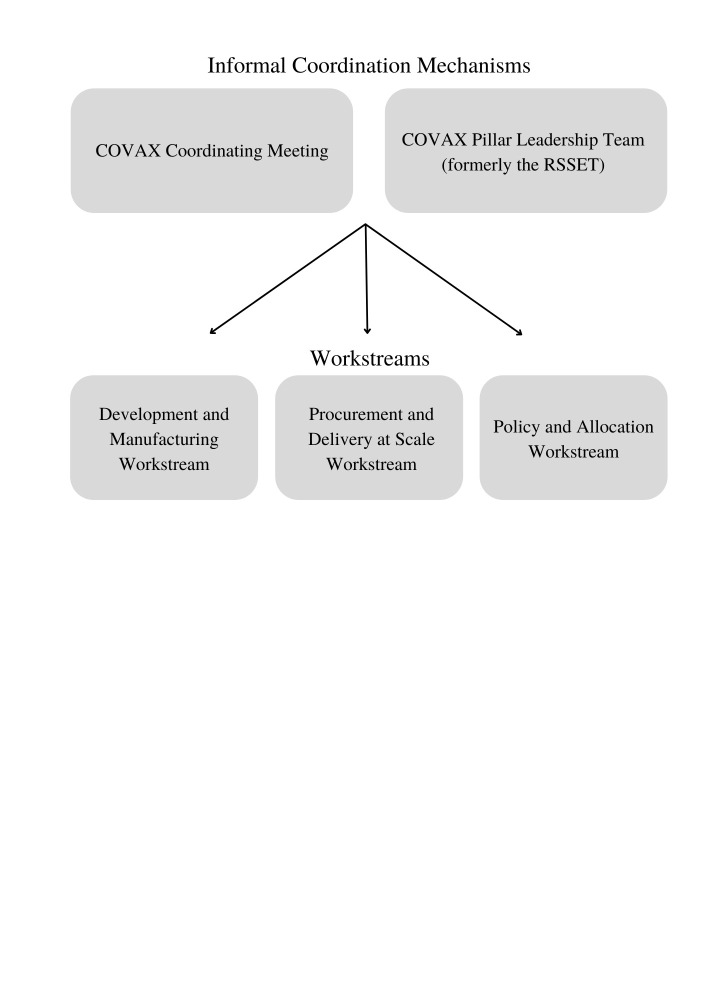
COVAX’s overarching governance mechanism.

COVAX’s workstreams were overseen and coordinated by two cross-cutting informal coordination mechanisms (COVAX participant 7) (**Figure 1**), two high-level informal governance bodies that spanned and were responsible for each of the three workstreams. The first of these mechanisms was the COVAX Coordinating Meeting *“which convene[d] the principles of the COVAX organizations with various external stakeholder”* (COVAX participant 7) and which involved COVAX’s co-convening organisations, civil society organisations, and the pharmaceutical industry. The COVAX Coordinating Meeting was chaired by the chairs of the Gavi and CEPI boards and was run in collaboration with civil society representatives and representatives from the pharmaceutical industry [15]. The individuals involved in the COVAX Coordinating Meeting met every two weeks for two hours to ensure that all the work done by each of COVAX’s three workstreams complied with the ACT Accelerator’s goals (to protect economic and health systems around the world by rapidly ending the acute phase of the COVID-19 pandemic [48]), to address barriers to the achievement of these goals, and to resolve disagreements between co-convening organisation when they arose [15,55].

The second informal coordination mechanism was the COVAX Pillar Leadership Team (**Figure 1**), previously called (and described by some interviewees from within COVAX co-convening organisations) as the RSSET, *“which stands for Richard, Seth, Soumya, Eva, and Ted”* (COVAX participant 2). According to an interviewee, these five individuals and their deputies *“[met] once a week to discuss… more strategic issues and decision-making”* related to COVAX (COVAX participant 7).

The data describing COVAX’s governance structure suggests that the partnership achieved the first premise of shared health governance: that *“(m)ultiple societal actors… engage in a joint enterprise that either by omission fails or by collective action succeeds in co-producing the conditions… for all to be healthy”* [22]. With COVAX, these conditions were the global equitable distribution of COVID-19 vaccines and ending the acute phase of the COVID-19 pandemic. Its co-convening organisations collaborated (albeit informally) to create a joint enterprise through which they contributed to the initiative’s goals, and which, in theory, by inaction would fail or by collective action would succeed in stimulating global COVID-19 vaccine equity; as such, COVAX employed the first premise of shared health governance.

The second premise of shared health governance is that global health actors commit to producing the conditions for all to be healthy. Prah Ruger explains that, to ensure that global health actors meaningfully commit to producing these conditions, individuals and/or organisations involved in a global health initiative must define the public moral norms that guide their work, agree on how actors should adhere to them, and decide what should happen when disagreements arise [22]. COVAX actors did this as each of its co-convening organisations committed to promoting vaccine equity (the public moral norm) and agreed to define vaccine equity as vaccinating a minimum of 20% of participating countries’ populations [13]; they also agreed on how each organisation could contribute to this goal. These organisations’ collaborative governance bodies and recurring meetings offered platforms for them to address disagreements and discuss how to ensure the partnership progressed smoothly; as such, COVAX employed the second premise of shared health governance.

Additionally, shared health governance is premised on shared commitments to an ideal [22] which can serve as motivation for actors’ contribution to a global health initiative and can bind the *“subsystems”* of the initiative together [22]. Within COVAX, this ideal was to end the acute phase of the pandemic by the end of 2021 and the co-convening organisations were the initiative’s subsystems. Ideals about how to end the pandemic (i.e. facilitate global equitable vaccine distribution) formed the basis of the organisations’ roles in the partnership, where each had a specific role to play and which bound each of them to their shared goal. Since each co-convening organisation in COVAX was governed by and responsive to its board, and given that the cross-cutting governance structures were all informal, the organisations’ commitments to each other appeared to be guided by individual constitutional commitments rather than to collective ones. Nonetheless, COVAX achieved this premise.

Shared health governance is based on the concept of shared sovereignty [22]. Specifically, when there is shared sovereignty, *“(d)uties are distributed according to the functional abilities of global, national, local, and individual actors and a comprehensive grasp of the actions needed to address global and domestic health problems”* [4]. Although each of COVAX’s co-convening organisations was individually governed, they were also all in charge of the components of the partnership in which they had previous experience (i.e. CEPI led vaccine research and development, UNICEF was in charge of procurement, etc.). Consequently, COVAX had shared sovereignty, so it met this premise of shared health governance.

COVAX’s achievement of shared health governance (premises one to three and six) suggests that COVAX’s co-convening organisations were committed to jointly running the partnership, divvying roles by which to contribute to its goals, and committing to its overarching goals. This offers insight into how global health initiatives comprised of a variety of international actors can collaborate to address a shared global health goal.

### Accountability

#### Formal accountability mechanisms

In COVAX, the governance structure in which each co-convening organization was governed by its own individual board meant that each co-convening organisation was formally accountable to its board as well. As an interviewee from a COVAX co-convening organisation explained:

*“…different areas have different accountabilities… so, in other words, on the allocation to countries that’s WHO that’s accountable for that… the sort of firm order contracts sits with Gavi... I think it’s relatively clear every organization’s role and within that, there are people who are accountable to specific areas. So if things come up for decision or discussion it’s clear this institution is responsible for shepherding this through… we don’t have one body where the accountability sits.”* (COVAX participant 3).

The structure in which each co-convening organisation is formally accountable to its board suggests that COVAX did not employ mutual collective accountability, as COVAX did not achieve the framework’s hallmark – that actors hold each other accountable for achieving a shared global health goal. Employing mutual collective accountability would require that every actor involved in COVAX was accountable to one another; that is, that they actively communicated with and reported to one another, shared responsibility for their actions and decisions, and collectively answered for their successes and failures.

#### Challenges with COVAX’s accountability

Interview participants explained that the accountability structure in which each co-convening organisation was formally accountable only to its board was ineffective and inefficient. As an interviewee explained:

*“when you have multiple governments and countries involved, it’s very difficult, I think, to have a crisp, clean [accountability]”* (COVAX participant 3). The interviewee elaborated that *“sometimes there’s grey areas where a decision crosses accountabilities, and that’s, I think, where we may lose something…when you get into those intersections that we run into sometimes a little bit of a challenge”* (COVAX participant 3).

It thus appears that although the formal governance structure in which each co-convening organisation was governed by its own board was necessary for COVAX to be established rapidly, this structure had serious implications for the efficacy of the partnership’s accountability mechanism.

#### Informal accountability mechanisms

Given the challenges with COVAX’s formal accountability structure, interviewees from within and external to COVAX agreed that much of the meaningful and impactful accountability for COVAX’s operations instead came from informal ones. These mechanisms were not established as part of the COVAX partnership, but developed from individuals’ desire for COVAX to be responsive to its stakeholders for its actions and decisions.

Specifically, interviewees explained that the most impactful form of informal accountability *“[came] down to the press”* (Journalist 3). An interviewee from one of COVAX’s co-convening organisations further explained that *“COVAX has been maybe not on the front page of the newspaper every day, but [they’ve] been subjected to intense media scrutiny for months. And I think that’s also been quite important in driving accountability”* (COVAX participant 7). In October of 2021, for example, the Bureau of Investigative Journalism published an article titled “How COVAX Failed on its Promise to Vaccinate the World” in which it exposed COVAX’s shortcomings, including its failure to deliver COVID-19 vaccines in a timely manner [74]; it does not appear, however, that this article prompted any action to be taken against the partnership.

Interviewees explained that informal accountability also came from within those involved in COVAX who felt a moral responsibility to *“…the vision… the goal… the equity agenda”* (COVAX participant 4). An interviewee explained that *“there’s more at stake than your individual actions, and it has implications across the board… And I think also just like literally getting the vaccines to people, like we count that”* (COVAX participant 4). Individuals who work for COVAX co-convening organisations, therefore, had a moral obligation to promote global equitable access to COVID-19 vaccines; thus, they held themselves accountable for achieving this goal.

COVAX actors’ moral commitments to contribute to the partnership’s goals are evidence that COVAX achieved the second premise of shared health governance – that global health actors commit to producing the conditions for global COVID-19 vaccine equity. As an informal accountability mechanism, the moral responsibility to contribute to COVAX’s goals created strong commitments to the partnership that functioned as motivation to ensure that its objectives were met. Data has not demonstrated, however, whether the internalisation of vaccine equity and moral responsibility for contributing to COVAX’s success led to *“…a shared authoritative standard by which individuals and groups can use their health agency to make more effective decisions for optimal individual and societal health”* – a component of the fifth premise of shared health governance [22]. Consequently, while COVAX actors did not face formal sanctions for failing to contribute to the partnership’s goals, we cannot conclude as to whether they faced social sanctions based on their internalisation of public moral norms.

### Transparency

COVAX had policies about transparency related to clinical trial data sharing, price transparency, and the sharing of COVID-19 vaccine delivery schedules. Clinical trial data related to the development and/or manufacturing of COVID-19 vaccines financed through CEPI, for example, were required to be made publicly available *“…to ensure that all can benefit from the work funded by CEPI”* [75]. Additionally, COVAX policy was *“…to communicate vital and timely information to a spectrum of COVAX stakeholders – documents relating to COVAX’s operations and allocation decisions are made available to the public via the Gavi website…”* [66].

Besides official policies for transparency, COVAX made several public calls-to-action to promote transparency in COVID-19 vaccine distribution. For example, COVAX publicly stated that its *“…goal [was] to maintain transparency, and the usual practice of UNICEF Supply Division is to publish prices unless a specific confidentiality agreement applies”* [66]. Similarly, COVAX partners made a public appeal for vaccine manufacturers to be more transparent about their vaccine delivery schedules and countries’ positions in their queues [59,76,77]. Interviewees from within COVAX’s co-convening organisations discussed how this was generally achieved, as public websites and dashboards provided information about vaccine deliveries and delivery forecasts [16].

#### Surface-level transparency

Other interviewees, however, expressed frustration at COVAX’s transparency, explaining that their policies and calls-to-action were insufficient and ineffective because they were seldom enforced. According to an interviewee, for example, even though there was some transparency about allocation decisions, COVAX’s allocation algorithm itself was *“top secret”* (Journalist 3). As a result, it was difficult for recipient governments to understand why they had (or had not) been allocated certain vaccines at specific times. This was significant, as it *“…hinder[ed] informed discussions about options for scaling up vaccine production, [made] it difficult to hold principal actors accountable, and prevent[ed] fair and equitable vaccine distribution”* [78].

The lack of access to this algorithm called into question whether COVAX achieved the fourth premise of shared health governance: that actors in a global health initiative share resources fairly and that aid recipients only take what they need. Without access to the algorithm used to make decisions, it is difficult to discern whether allocations were based on countries’ needs, and thus, whether they met this premise of shared health governance. This is significant, as understanding how allocations are made is crucial for holding decision-making bodies to account for their role in vaccine deliveries, and in turn, in stimulating global COVID-19 vaccine equity.

Concerning public access to information, interviewees external to COVAX qualified the utility of COVID-19 vaccine distribution websites and dashboards by explaining that the information they provided was sometimes inaccurate and/or incomplete. As a participant from an NGO described:

*“…directly speaking with Gavi at meetings on Zoom obviously, and saying… where is this particular piece of information, they’re saying ‘oh that’s out there’… then the website that they referred to is the website that I’ve got open and I’m looking at it and like yes, it has some of the information I’m talking about, but it does not have all the information I’m asking for. It has a column for it, but there’s no numbers in that column”* (NGO participant 9).

This demonstrates a significant difference in the way interview participants from within COVAX co-convening organisations and those external to the partnership perceived COVAX’s transparency. While most participants agreed that there were publicly available platforms for accessing information about the partnership, they disagreed about their utility, completeness, and openness. Thus, while interview participants from within COVAX believed the partnership to be sufficiently transparent, those external to it generally believed transparency to be lacking.

#### Protecting stakeholder interests

Many participants, regardless of how they perceived and discussed COVAX’s transparency, acknowledged that the partnership had limitations in what types of information it could share publicly. Notably, in 2021 and into 2022, COVAX did not provide public access to the Facility’s contracts with COVID-19 vaccine manufacturers. Most vaccine purchasing contracts contained confidentiality clauses that prohibited their publication. According to one participant:

*“…when a lot of these deals were signed, there were no vaccines, there was no kind of pandemic response, and so the manufacturers were in a hugely powerful position and basically got to, not quite determine the terms they wanted, but the deals were pretty one-sided. And that was just buyer power versus supplier power…and some of those deals included lots of confidentiality clauses” (*COVAX participant 2).

As a result of confidentiality agreements that hindered contract transparency, most interviewees explained that prices paid per vaccine dose were obscured in 2021 and early 2022. However, in March of 2022, UNICEF and Gavi published the prices of COVAX-procured vaccines, with some exceptions [33] (COVAX participant 7). When prompted about why certain exceptions for the disclosure of prices were granted, an interview participant from a COVAX co-convening organisation stated that they could not provide details, suggesting a further lack of transparency.

Interview participants explained that manufacturers included confidentiality clauses in their contracts with COVAX to protect their commercial interests. Obscuring vaccine prices limited COVAX’s ability to engage in informed negotiations with manufacturers, and thus, to advocate for the lowest price possible. Thus, participants who did not work for a COVAX co-convening organisation expressed frustration at COVAX’s lack of transparency on vaccine prices, explaining that without access to prices, *“[manufacturers could] use the lack of knowledge or the knowledge asymmetries to reap high prices”* (NGO participant 5).

In 2021, it was estimated that the cost of producing an mRNA COVID-19 vaccine was approximately US$1.18 per dose [79]. COVAX, however, had reportedly been paying close to five times that amount, while national governments procuring vaccines bilaterally had paid between four and 24 times that amount [79]. Due to the lack of pricing transparency and the demand for inflated prices per dose, COVAX was pushed behind high-income countries in the vaccine delivery queue, as they were willing and able to spend more than COVAX to procure COVID-19 vaccines [79]. There is also evidence that COVID-19 vaccine manufacturers offered different prices per vaccine dose to different countries, though not by what would be expected based on countries’ gross domestic products; this contributed to inequities in vaccine distributions as well [27].

While describing fair resource sharing (shared health governance premise #4), Prah Ruger explains that “we all share in the benefits that accrue to society from achieving justice in health, including a more healthy, stable, well-cared-for, productive population, as well as cost containment and reduction in disease risk. Thus, we all share in mobilising and using the resources necessary to achieve this end” [22]. Our data demonstrates that vaccine manufacturers did not contribute to vaccine cost containment for doses procured through COVAX, as contract opacity permitted them to increase prices and maximise profits. This further suggests that COVAX did not facilitate fair resource sharing and thus failed to achieve the fourth premise of shared health governance.

### COVAX’s shortcomings

COVAX did not achieve its initial goal of distributing two billion doses of COVID-19 vaccines by the end of 2021 [74,80-83]. Interviewees from within and external to COVAX were forthcoming in their discussions about COVAX’s shortcomings. As one interviewee explained, *“COVAX has not been a big success. We aimed for 2 billion doses by the end of the year [2021], we got to 950 million. That’s under 50% of the target”* (COVAX participant 2).

We were able to examine why COVAX may have had difficulty achieving its goals. First, COVID-19 vaccine manufacturers received patent protection for their products, through which they limited the global supply of these vaccines [84,85]. In October 2020, the Indian and South African governments initiated a campaign for intellectual property (IP) waivers for COVID-19 vaccines. While this campaign was eventually supported by more than 100 countries [84], COVAX did not take a firm stance on IP waivers, instead stating that “*[n]egotiations between governments and manufacturers around intellectual property… [were] not in the purview of the work of COVAX”* [66]. According to Tamaryn Nelson of Amnesty International, however, *“(b)y refusing to engage with initiatives that have the potential to significantly boost global vaccine supply, COVAX seem[ed] to be shooting itself in the foot and hampering its very own work”* [86]. A participant from an NGO echoed this sentiment by asserting that:

*“I personally think that COVAX was destined to fail from the get go. And it’s not because it [was] badly designed, it’s not because there wasn’t motivation or there wasn’t the funding that was needed… I think that the thing that held it back was patent protection”* (NGO participant 3).

Additionally, interviewees agreed that vaccine manufacturers’ prioritisation of bilateral deals with high-income countries over their contracts with COVAX hindered COVAX’s success. As one interviewee asserted, this resulted in COVAX *“working with one hand tied behind their back”* (Journalist 1). For example, by October of 2021, COVAX was responsible for only 5% of the COVID-19 vaccines distributed around the world, leaving 98% of the global population without access to a first or second dose of a vaccine [87]. This, according to Transparency International, led to a *“‘two-track pandemic,’ with high vaccination rates and a lifting of restrictions in economically powerful countries, while the rest of the world continue[d] to battle wave after wave of infections”* [87].

While many interview participants external to COVAX described this *“two-track pandemic”* as unfair and immoral, an interviewee from within a COVAX co-convening organisation instead explained that:

*“…in the grand scheme of fairness, is it fair that high income countries got their doses first? Arguably yes, because they actually stumped up the money. Is it fair that low income countries didn’t have the money to stump up? No. That’s the result of colonialism, huge amounts of structural injustice… [between countries]”* (COVAX participant 2).

Data suggests that the entirety of COVAX, however, was premised on the belief that there would be a collaborative global response to the pandemic, in which countries would work together to promote global equitable vaccine distribution. This, however, ignored the deeply embedded nationalism and colonialism that so powerfully impact global health. Accordingly, interviewees from within and external to COVAX agreed that COVAX was *“naïve”* and *“…too candid in thinking that solidarity [would] be enough…”* (COVAX participant 1). One interviewee asserted that COVAX was “*…overly ambitious to think that you could get a kind of global response to something that there [was] so much kind of domestic interest in as well…”* (NGO participant 4).

Data also suggests that COVAX’s lack of transparency contributed to its shortcomings. For example, one participant asserted that COVAX’s lack of transparency hindered the ability of recipient countries to absorb and deploy COVID-19 vaccines because *“it [was] hard to plan a vaccination campaign when you [did not] know what vaccines [were] coming and when”* (NGO participant 9). Thus, when vaccines were delivered, countries were unable to use them rapidly and efficiently, leading to high volumes of vaccine wastage [56,74,88].

Furthermore, the general lack of transparency in COVAX made it difficult to hold actors involved in vaccine distribution to account for their actions and decisions [78]. As an interviewee explained, COVAX’s lack of *transparency “…made it difficult to conduct public monitoring and hold pharmaceutical companies accountable when they fail[ed] to meet their commitments”* [78]. Additionally, a participant explained that the lack of transparency about vaccine donations meant that it was *“almost impossible to hold the [donating] country to account for those decisions…That is an example of why transparency is important in terms of accountability”* (NGO participant 5). Without the ability to hold COVAX actors to account for their actions and decisions, it was difficult to motivate these actors to contribute to its goals.

## DISCUSSION

Our findings indicate that COVAX achieved most but not all the premises of shared health governance. The facets of its governance that met the first three and last premise of shared health governance – multiple societal actors engaging in a joint enterprise, a commitment to producing the conditions for all to be healthy, shared commitments to an ideal, and shared sovereignty – may have benefitted the partnership and may be worth including in future large-scale global health initiatives like COVAX. That said, future research should evaluate whether these components of the partnership did benefit its operations and if so, in what way.

Although data has not provided evidence as to whether the partnership involved social sanctions for failure to contribute to its goals, COVAX did not promote fair resource sharing. The partnership, therefore, did not have the necessary foundation for shared health governance. Our analysis also suggests that COVAX did not employ mutual collective accountability and was inadequately transparent. These findings are in line with existing literature about the lack of transparency in COVAX’s operations [34,36], its complex and flawed governance structure [34,35], and the importance of good governance, accountability, and transparency in the global response to the COVID-19 pandemic [28-33] by analysing the implications that ineffective governance, accountability, and transparency have for health equity.

Participants’ willingness to both critique COVAX and acknowledge its successes highlights that there are lessons to be learned from the partnership’s accomplishments and the challenges it faced. Among these lessons is the one on the role of the pharmaceutical industry and vaccine manufacturers in global health initiatives. While COVID-19 vaccine manufacturers were undeniably in a position of power, this study calls into question the role that this power should play during public health crises. As many interview participants explained, COVID-19 vaccine manufacturers had great influence over vaccine procurement contracts and the prices paid per vaccine dose. This hindered global equitable access to COVID-19 vaccines by limiting the possibility for the COVAX Facility to engage in informed negotiations and by inflating vaccine prices.

While our findings do not provide concrete mechanisms by which COVAX could have addressed these power asymmetries, they do offer insight into the types of mechanisms that are conducive to – and arguably promote – power imbalances between the pharmaceutical industry and recipients of their products. For example, COVAX’s failure to take a stance on IP protections and their hope that there would be a collaborative global response to the pandemic essentially enhanced pharmaceutical companies’ powers. COVAX was premised on the belief that countries, the pharmaceutical industry, and international organisations would authentically collaborate to promote the global equitable distribution of COVID-19 vaccines. Without mechanisms to incentivise this collaboration, however, individual national and commercial interests were permitted to supersede those of the global interest. As such, we argue that promoting equitable and mutually beneficial global collaboration for health requires the active implementation of policies that incentivise and/or promote meaningful collaboration between actors.

This study confirms existing accounts of power asymmetries between pharmaceutical companies and the recipients of their products [71,89] and underscores the need to adopt new public health systems that better serve global and public interests. Mazzucato and Li, for example, propose reducing these power asymmetries by conducting a “…reframing of the role of the state in innovation from market-fixing to market co-creation and co-shaping, in which risks and rewards are shaped across a symbiotic public-private relationship” [89]. While our study does not provide evidence as to whether this would have worked in COVAX, it underscores the need for future research that explores how to overcome these power imbalances in large global health initiatives like COVAX.

Our study also calls into question the feasibility of authentic global collaboration for the achievement of a shared global health goal. Rosenberg et al. [57] assert that “(c)ollaboration, never more essential than today, has also never been more challenging”; the COVID-19 pandemic significantly exacerbated this need and these challenges. We currently live in a global society where the individual interests of the most powerful (i.e. vaccine manufacturers and high-income countries) are permitted to supersede those of the global community. Our study thus corroborates existing accounts of difficulties in achieving authentic global collaboration to address global health challenges [57] and highlights that the COVID-19 pandemic increased the urgency with which these challenges must be addressed.

### Limitations

This study had a few limitations. First, all interviews were conducted in English. Given that COVAX was a global initiative, it is likely that there were individuals well-suited to participate in this study who were not recruited because of language barriers. Furthermore, interview recruitment was conducted during the Omicron wave of the COVID-19 pandemic. Given the large toll this wave took on health systems around the world [2], many potential interview participants who were involved in COVAX explained that they could not participate because they were too busy assisting countries in their responses. Several other participants agreed to participate in the study, but were unable to dedicate enough time to providing detailed information to all the interview questions. It is possible that this limited both the breadth and depth of data collected from interview participants for this study.

Furthermore, we did not conduct an in-depth analysis of all facets of vaccine equity. Although some participants spoke briefly about the role of IP and IP waivers on COVAX’s ability to achieve its goals, most participants were rather dismissive of these topics. Although this might have reflected the types of individuals who participated in interviews (people in the advocacy and global health spheres rather than individuals who work in the pharmaceutical industry), this presented a gap which should be addressed in future studies.

Lastly, by using a meaning saturation methodological approach, it is possible that we missed important data. Given the rapidly-evolving nature of the subject and in the interest of efficiency, we chose to end data collection at meaning saturation, which means that we stopped collecting new data when successive interviews of individuals of the same group (those internal to COVAX and those external to it) no longer provided new information. Other studies have come to similar conclusions about the weaknesses in the partnership’s governance, transparency, and accountability [34-36]; further studies would have to be conducted, however, to determine whether we have collected all important data about COVAX as it relates to shared health governance and mutual collective accountability.

## CONCLUSIONS

Here we explore the mechanisms for governance, transparency, and accountability in COVAX and assess their compliance with the shared health governance and mutual collective accountability frameworks. Our findings suggest that COVAX only achieved four of the six premises of shared health governance. Most notably, it did not stimulate fair resource sharing between actors involved in the partnership and thus did not employ shared health governance. Additionally, while COVAX achieved each of the three requirements of mutual collective accountability, actors involved in the partnership did not hold one another accountable for their role in stimulating global COVID-19 vaccine equity; COVAX, therefore, did not employ mutual collective accountability.

The fact that COVAX did not employ shared health governance and mutual collective accountability does not fully explain its shortcomings, but does highlight weaknesses in its governance, accountability, and transparency, that, if addressed, could have improved its ability to achieve its goals. These results thus underscore the important role that good governance, accountability, and transparency play in the global response to the COVID-19 pandemic and offer lessons to be learned for the operation of future similar global health initiatives.

## Additional material


Online Supplementary Document
