# Supplementary material for: Shared health governance, mutual collective accountability, and transparency in COVAX: A qualitative study triangulating data from document sampling and key informant interviews
Source: J Glob Health. 2023 Dec 8;13:04165. doi: 10.7189/jogh.13.04165 (PMC10705035; doi:10.7189/jogh.13.04165)
Supplement: Online Supplementary Document [file jogh-13-04165-s001.pdf]

File S1: COREQ guidelines

No. Item	Guide questions/description	For this study
Domain 1: Research team and reflexivity		
1. Interviewer/facilitator	Which author/s conducted the interview or focus group?	AG conducted all interviews.
2. Credentials	What were the researcher's credentials?	AG is a master's student with research experience in health policy and training in qualitative methods,
3. Occupation	What was their occupation at the time of the study?	AG is a master's student with research experience in health policy and training in qualitative methods.
4. Gender	Was the researcher male or female?	AG is an English-speaking white female.
5. Experience and training	What experience or training did the researcher have?	AG is a master's student with research experience in health policy and training in qualitative methods.
6. Relationships established	Was a relationship established prior to study commencement?	No relationship was established prior to commencement.
7. Participant knowledge of the interviewer	What did the participants know about the researcher?	All participants were told AG's credentials and the overall purpose of the study.
8. Interviewer characteristics	What characteristics were reported about the interviewer/facilitator?	All participants were told AG's credentials and the overall purpose of the study.
Domain 2: study design		
9. Methodological orientation and Theory	What methodological orientation was stated to underpin the study?	We used a generic qualitative approach grounded in a critical realist perspective.
10. Sampling	How were participants selected?	We recruited interview participants using purposive and snowball sampling.
11. Method of approach	How were participants approached?	Participants were approached via email.
12. Sample size	How many participants were in the study?	There were 20 interview participants in this study.
13. Non-participation setting	How many people refused to participate or dropped out? Reasons?	21 individuals did not respond to recruitment emails and 22 individuals stated that they could not participate. Among the reasons for declining an interview request was lack of time, not being at

		liberty and/or in the position to discuss governance, transparency, and/or accountability in COVAX, or the presence of confidentiality/non-disclosure agreements with their employers that precluded them from speaking with us.
14. Setting of data collection	Where was the data collected?	Interviews were conducted over Zoom.
15. Presence of non-participants	Was anyone else present besides the participants and researchers?	No.
16. Description of sample	What are the important characteristics of the sample?	Of the 20 participants, 8 were employees of COVAX co-convening organizations (or individuals otherwise involved in COVAX), 9 were NGO employees, and 3 were journalists. Interview participants represented individuals from each of COVAX's co-convening organizations, 3 media outlets, and 7 NGOs. Nine participants were located in North America (45%), 8 in Europe (40%), two in Africa (10%), and 1 in South America (5%).
17. Interview guide	Were questions, prompts, guides provided by the authors? Was it pilot tested?	We sent participants interview questions in advance if explicitly requested. The interview guide was modified as necessary throughout the interview process.
18. Repeat interviews	Were repeat interviews carried out? If yes, how many?	Repeat interviews were not carried out.
19. Audio/visual recording	Did the research use audio or visual recording to collect the data?	If consent was provided, interviews were audio recorded.
20. Field notes	Were field notes made during and/or after the interview or focus group?	General field were not taken during or after interviews.

21. Duration	What was the duration of the interviews or focus group?	Interviews were between 20 and 45 minutes in duration (mean = 31 minutes).
22. Data saturation	Was data saturation discussed?	We stopped recruitment 4 interviews after we had reached meaning saturation. We determined that we reached meaning saturation when successive interviews of individuals of the same group (those internal to COVAX and those external to it), no longer provided new information; this occurred after 5 interviews with informants involved in the partnership and 12 interviews with informants external to COVAX. After saturation had been reached for each group, we interviewed 1 more individual external to COVAX and 3 more individuals from within co-convening organizations.
23. Transcripts returned	Were transcripts returned to participants for comment and/or correction?	Transcripts were not returned to participants for comment and/or correction.
Domain 3: analysis and findings		
24. Number of data coders	How many data coders coded the data?	One coder coded the interview data.
25. Description of the coding tree	Did authors provide a description of the coding tree?	AG wrote a memo containing a descriptive summary of the data under the code and an interpretation of these data stemming from the theoretical framework. JCK and QG read and commented on the memos to provide additional interpretation and to validate interpretation.
26. Derivation of themes	Were themes identified in advance or derived from the data?	We identified themes in advance.
27. Software	What software, if applicable, was used to manage the data?	We used NVivo 12 qualitative coding software.

28. Participant checking	Did participants provide feedback on the findings?	Participants did not provide feedback on the findings.
29. Quotations presented	Were participant quotations presented to illustrate the themes/findings? Was each quotation identified?	Participant quotations are presented in the manuscript to illustrate themes/findings. Each quotation was identified with participants' categories (COVAX, NGO, or reporter) and participant numbers.
30. Data and findings consistent	Was there consistency between the data presented and the findings?	There was consistency between the data presented and the findings.
31. Clarity of major themes	Were major themes clearly presented in the findings	Major themes were clearly presented in the findings.
32. Clarity of minor themes	Is there a description of diverse cases or discussion of minor themes?	This manuscript presents major themes (the manuscript has sub-sections for major themes).

File S2: Interview Guide

Hi, and thank you so much for coming, I appreciate you taking the time. I'm Ariel, a Master's student in the Leslie Dan Faculty of Pharmacy at the University of Toronto. Before we begin I'm going to go through some information about consent. As a brief reminder, the purpose of this study is to explore governance, transparency, and accountability in COVAX. Your participation in this study is entirely voluntary and you can refuse to participate or withdraw from the interview at any time. When you leave the interview, you can decide whether you want us to keep your data or not, you can just email me to let me know. You can also withdraw your data at any time prior to data analysis, which will start in March, which is when all the information will be anonymized so I won't be able to get rid of your data specifically any more. Please also note that your choice to participate will not influence your future relations with the University of Toronto or the WHO Collaborating Centre for Governance, Accountability, and Transparency in the Pharmaceutical Sector. Before I go on, do you have any questions?

Based on your consent form, the [audio will/won't be recorded]. If it's ok with you I will start the recording now. OR during the interview I will be taking notes to write down the information we discuss.

1) Can you please tell me about how COVAX is setup/governed?
    a. Can you tell me about the roles of the individual organizations within COVAX (Gavi, WHO, CEPI, UNICEF) in COVAX?
    b. Can you describe whether there is any collaboration between the organizations?
2) Can you tell me about some of the accountability mechanisms within COVAX (mechanisms to make sure that those who make decisions for COVAX are held to account for those decisions)?
    a. If there are accountability mechanisms, can you explain their efficacy?
    b. Are there consequences for not contributing to COVAX's mission? If so, what are they? If not, what seem to be the implications of not having consequences?
3) Can you please describe funding structures in COVAX? For example, how are resources (i.e. money, vaccines, syringes, etc.) distributed within COVAX?
    a. Have resources been shared fairly among COVAX actors and among those involved in COVAX/COVAX-participating economies? Explain.
4) Describe transparency within COVAX (i.e. transparency about vaccine distributions and allocations, prices per dose, transparency between vaccine manufacturers and COVAX officials, national governments, etc.)
5) In your opinion, has COVAX achieved its goal of promoting global vaccine equity and of prioritising global over national interests?
6) Is there any information that you feel is relevant that you would like to add to this discussion?
7) Can you please provide me with names of individuals who could also provide me with information on the topics of transparency, accountability, and governance in COVAX?

Those are all the questions I have for you. Do you have any questions you would like to ask me before we wrap up?

Thank you so much for taking the time to participate in this study. If you have any questions or concerns related to the study or your interview, please feel free to email me on the same email we set up the interview

File S3- Coding Framework

Code	Definition
1. COVAX goals, achievements, and decisions	COVAX's achievements to date (i.e. how many doses they have delivered); COVAX's specific goals (i.e. vaccinate 20% of the world against COVID-19); information about COVAX's vaccine distributions to date and/or their distribution projections; goals such as commitment to promoting health/vaccine equity; global equitable distribution of COVID-19 vaccines
2. COVAX failures, barriers to implementation, and challenges	Failures to achieve COVAX's goals; challenges that have arisen and have made it difficult to meet COVAX's goals, etc. Include information about risks to COVAX's success (i.e. indemnification clauses that have made it difficult for countries to sign contracts).
3. Governance	General information about governance structures within COVAX (i.e. who does what role within the Facility), including suggestions for good governance. Also include information about who makes certain decisions within the Facility (i.e. the JAT makes vaccine allocation decisions).
4. Accountability	General information about accountability in COVAX. Definition of accountability: "…actors have the right to hold other actors to a set of standards, to judge whether they have fulfilled their responsibilities in light of these standards, and to impose sanctions if they determine that these responsibilities have not been met" (Grant and Keohane, 2005). Include information about auditing, investigations, and oversight mechanisms (i.e. the IAVG conducts oversight of the JAT's vaccine allocation decisions).
5. Transparency	General information about transparency within COVAX including allocation decisions, COIs, data, distribution, funding, price per dose, etc. Include calls for increased transparency within the Facility or information about the lack of transparency and any examples of transparency (i.e. transparency in vaccine allocation decisions).

6. Collaboration	Evidence or examples of global health actors collaborating within COVAX (i.e. WHO, UNICEF, Gavi, CEPI, CSOs working together, etc.).
7. Sharing resources	Any information about resource-sharing within COVAX. Resources include COVID-19 vaccines, clinical trial data, funding for vaccines and ancillary products, etc. Code whether/how actors contribute resources.
8. Social sanctions	Any evidence of social sanctions imposed if actors deviate from COVAX's goals. Include enforcement of these sanctions.
9. IP	Any discussion about IP and information about the need for IP waivers to promote global COVID-19 vaccine equity
10. Decision-making processes	Any information about how decisions are made within COVAX (i.e. the factors on which decisions are predicated such as the WHO SAGE's values framework for the distribution of vaccines). This is distinct from code 5 (governance) in that it does not include information about *who* makes decisions. Include information about COVAX's risk appetite.

File S4- Documents/webpages included in document analysis

Source	Title	Publication date
CEPI	CEPI opens search for experts to join its Scientific Advisory Committee	January 25, 2021
	COVAX Manufacturing Task Force to tackle vaccine supply challenges	May 14, 2021
	COVAX: CEPI's response to COVID-19	November 18, 2020
	Enabling Equitable Access to COVID-19 Vaccines: Summary of equitable access provisions in CEPI's COVID-19 vaccine development agreements	March 18, 2021
	Plague Inc. The Cure: Behind the scenes	2021
	CEPI launches COVAX Marketplace to match buyers and sellers of critical manufacturing supplies and speed up global access to COVID-19 vaccines through COVAX	July 15, 2021
	Joint COVAX statement on Supply Forecast for 2021 and early 2022	September 8, 2021
	Vaccine specialists and global health experts selected for CEPI's Scientific Advisory Committee	June 9, 2021
COVAX (joint publication between COVAX's co-convening organizations)	Manufacturing Taskforce	May 12, 2021
	Dialogue with civil society: ACT-A and COVID-19 vaccines	October 27, 2020
	Briefing Note: Additional Information on Cost Sharing for COVAX AMC Participants	November 2020
	COVAX Facility convenes first meeting of COVAX Shareholders Council	November 3, 2020
	COVAX Facility governance explained	November 13, 2020
	COVAX: Policy and Allocation of vaccines: update, status and next steps	n.d.
	COVID-19 Vaccine Access: Response to Joint Letter from Human Rights Watch, Public Citizen, MSF Access Campaign and Amnesty International	March 25, 2021
	COVAX welcomes appointment of civil society representatives	October 30, 2020
	COVAX AMC Engagement Group Operating Procedures	February 2021
	COVAX Vaccine Request: Gavi Grant Terms and Conditions for COVAX AMC Group Participants	n.d.
	COVAX Independent Allocation of Vaccines Group	December 18, 2020
	COVAX Facility Shareholders Council Operating Procedures	November 2020
	COVAX Global Supply Forecast September 8, 2021	September 8, 2021

	Vaccine Request Annex A: COVAX Facility Terms and Conditions for the AMC Group Participants	n.d.
	COVAX Facility Explainer: Participation Arrangements for Self-Financing Economies	n.d.
	COVAX: The Vaccines Pillar of the Access to COVID-19 Tools (ACT) Accelerator Structure and Principles	November 9, 2020 (updated March 17, 2021)
	The COVAX Facility and the AMC DCVMN Annual General Meeting	November 4, 2020
	Guidance Note for Technical Assistance Support for Gavi-57 Economies' Preparedness and Readiness for COVID-19 Vaccine Delivery	n.d.
	One World Protected: The Gavi COVAX AMC Investment Opportunity	April 15, 2021
	ACT-Accelerator COVAX Pillar – Independent Product Group	n.d.
	COVAX Facility Terms and Conditions for Self-Financing Participants	n.d.
	The COVAX Humanitarian Buffer Explained	March 30, 2021
	COVAX AMC Engagement Group Terms of Reference	March 2021
	COVAX Facility Shareholders Council Terms of Reference	December 2020
	COVID-19 Vaccine Access: Response to Joint Letter from Human Rights Watch, Public Citizen, MSF Access Campaign, and Amnesty International	January 6, 2021
	Principles for Sharing COVID-19 Vaccine Doses with COVAX	December 18, 2020
	Briefing Note for Governments and Organisations: Secondments to the Office of the COVAX Facility	November 2020
	COVAX Pillar Strategy for 2022 and beyond	September 2021
Gavi	Gavi Alliance Board Meeting Minutes	September 29-30, 2020
	Gavi Alliance Board Meeting Minutes	December 15-17, 2020
	Gavi Alliance Board Meeting Minutes	March 22, 2021
	Gavi Report to the Board Annex A: Implications/Anticipated impact	December 3, 2021
	Gavi Report to the Board Annex C: COVAX Risk Report	December 6, 2021
	Gavi Report to the Board Annex D: COVAX Country Participation Model: Risk Considerations	December 6, 2021
	Gavi Report to the Board: Audit and Finance Committee Chair Report	June 23-24, 2021
	Gavi Report to the Board: Report of the Chief Executive Officer	June 23-24, 2021
	Report to the Gavi Board: IFFIM Chair Report	June 23-24, 2021

	Gavi Report to the Board: Report of Audit and Investigations	June 23-24, 2021
	Gavi Report to the Board 23-24 June 2021: COVAX Update	June 23-24, 2021
	Gavi Report to the Board 28 September 2021: COVAX: Key Strategic Issues, Report to the Board	September 28, 2021
	COVAX Facility Operationalisation and Vaccine Programme: Gavi Board Meeting	December 15-17, 2020
	92 low- and middle-income economies eligible to get access to COVID-19 vaccines through Gavi COVAX AMC	July 31, 2020
	Gavi Risk Appetite Statement Version 3.0	n.d.
	Gavi and humanitarian agencies partner to deliver COVID-19 vaccines to the most vulnerable people in the world	November 16, 2021
	Gavi Board meets to discuss routine immunization, COVAX's 2022 strategy	September 29, 2021
	Gavi Board strengthens commitment to reaching the most vulnerable through routine immunization and COVAX	June 25, 2021
	Consultancy Opportunity COVAX Technical Officer, Joint Allocation Taskforce- Dose Sharing	December 1, 2021
	The Vaccine Alliance: Risk and Assurance Report 2020	2020
	Reporting concerns of wrongdoing to Gavi and IFFIm	2020-2021
	Board and Committee Operating Procedures	June 2020
UNICEF	COVAX Facility News: UNICEF Partners with Airlines and International Carriers to Plan Delivery of COVID-19 Vaccines	November 23, 2020
	COVAX Joint Statement: Call to action to equip COVAX to deliver 2 billion doses in 2021	May 27, 2021
	Delivering COVAX supplies during supply chain crisis, the HOPE Consortium steps up support to UNICEF	October 7, 2021
	Leading airlines commit to helping UNICEF in its historic mission of transporting COVID-19 vaccines around the world	February 15, 2021
	Procurement of COVID-19 Vaccines for delivery in 2021	October 30, 2020
	Remarks by Henrietta Fore, Executive Director of UNICEF, at a briefing on COVAX's interim distribution forecasts	February 4, 2021
	UNICEF Executive Director Henrietta Fore's remarks at briefing on COVAX publishing interim distribution forecast: As delivered	February 3, 2021
	UNICEF Executive Director Henrietta Fore's remarks at the World Immunisation & Logistics Summit, hosted by the HOPE Consortium	March 29, 2021
	The historic push to provide ultra-cold chain freezers around the world	September 21, 2021
	The UNICEF Humanitarian Airfreight Initiative: Launched February 2021	February 16, 2021
	Responding to COVID-19: UNICEF's 2020 key achievements	April 2021

	UNICEF and PAHO launch joint COVID-19 vaccine tender on behalf of COVAX Facility	November 11, 2020
	UNICEF and the World Economic Forum sign charter with 18 shipping, airlines and logistics companies to delivery COVID-19 vaccines	December 21, 2020
	Responding to COVID-19: UNICEF Annual Report 2020	June 2021
	UNICEF fund aims to raise US$2.5 billion for COVID-19 health supplies; low- and middle-income countries set to benefit	March 30, 2021
	UNICEF to lead procurement and supply of COVID-19 vaccines in world's largest and fastest ever operation of its kind	September 3, 2020
	UNICEF to stockpile over half a billion syringes by year end, as part of efforts to prepare for eventual COVID-198 vaccinations	October 19, 2020
	Urgent action needed now to ensure sufficient COVID vaccine syringe supply to meet 2022 vaccination targets	October 27, 2021
	COVAX Update: UNCIEF working with global airlines and freight providers to plan delivery of COVID-19 vaccines	November 23, 2020
	COVAX: ensuring global equitable access to COVID-19 vaccines	n.d.
	UNICEF begins shipping syringes for global COVID-19 vaccine deployment as part of COVAX	February 23, 2021
	UNICEF outlining plans to transport up to 850 tonnes of COVID-19 vaccines per month on behalf of COVAX, in 'mammoth and historic' logistics	December 18, 2020
	Pricing data	September 21, 2021
WHO	Achieving 70% COVID-19 Immunization Coverage by Mid-2022: Statement of the Independent Allocation of Vaccines Group (IAVG) of COVAX	December 23, 2021
	What is the Access to COVID-19 Tools (ACT) Accelerator, how is it structured and how does it work?	April 6, 2021
	Joint COVAX statement on Supply Forecast for 2021 and early 2022	September 8, 2021
	Global leaders commit further support for global equitable access to COVID-19 vaccines and COVAX	September 23, 2021
	ACT-Accelerator partnership welcomes leadership and commitments at US COVID Summit to ending COVID-19 pandemic through equitable access to tests, treatments, and vaccines	September 24, 2021
	Strategic Plan & Budget October 2021 to September 2022: Enhancing equity in access to COVID-19 tools	October 28, 2021
	ACT-Accelerator Prioritized Strategy & Budget for 2021	April 12, 2021
	WHO SAGE values framework for the allocation and prioritization of COVID-19 vaccination	September 14, 2020
	Allocation logic and algorithm to support allocation of vaccines secured through the COVAX Facility: Explainer based on commonly asked questions	February 15, 2021

	COVAX Independent Allocation of Vaccines Group	December 18, 2020
	Report of the Independent Allocation of Vaccines Group on the allocation of COVAX Facility secured vaccines	July 29, 2021
	Joint Statement of the Multilateral Leaders Taskforce on Scaling COVID-19 Tools: A Crisis of Vaccine Inequity	August 27, 2021
	UN welcomes nearly $1 billion in recent pledges – to bolster access to lifesaving tests, treatments and vaccines to end COVID-19	September 30, 2020
	What Needs to Change to Enhance Covid-19 Vaccine Access: Statement from the Independent Allocation of Vaccines Group of COVAX	September 24, 2021
	WHO calls on world leaders at the UN General Assembly to focus on vaccine equity, pandemic preparedness, and getting the SDGs back on track	September 17, 2021
	COVID-19 immunization in refugees and migrants: principles and key considerations: Interim guidance	August 31, 2021
	Global leaders commit further support for global equitable access to COVID-19 vaccines and COVAX	September 23, 2021
	Fair allocation mechanism for COVID-19 vaccines through the COVAX Facility	September 19, 2020
Other: Transparency International	Another Shot at Vaccine Transparency	October 22, 2021
Other: Transparency International	COVID-19 Vaccine Transparency	March 2, 2021
Other: Transparency International, WHO Collaborating Centre for Governance, Accountability and Transparency in the Pharmaceutical Sector, University of Toronto Leslie Dan Faculty of Pharmacy	For Whose Benefit? Transparency in the development and procurement of COVID-19 vaccines	2021

Other: Human Rights Watch	COVAX: Enhance Transparency, Share Intellectual Property: Global Vaccine Initiative Should Fulfill Its Human Rights Responsibilities	May 6, 2021
Other: Health Cluster	Global Health Cluster Position on COVID-19 vaccination in Humanitarian settings: 23 key messages for advocacy	April 2021
Other: The Bureau of Investigative Journalism	How COVAX Failed on its Promise to Vaccinate the World	October 10, 2021
Other: World Bank Group	International Organizations, Manufacturers Agree to Intensity Cooperation to Deliver COVID-19 Vaccines	September 16, 2021
Other: World Bank Group	Joint Statement of the Multilateral Leaders Task Force on Scaling COVID-19 Tools	November 9, 2021
Other: African Union and Africa CDC	Joint Statement on Dose Donations of COVID-19 Vaccines to African Countries	November 29. 2021
Other: U4 Anti-Corruption Resource Centre	Global access to Covid-19 vaccines: Lifting the veil of opacity	2021
Other: U4 Anti-Corruption Resource Centre and Transparency International	Mitigating corruption risks in COVID-19 vaccine rollout: What can donors do?	April 7, 2021
Other: Transparency International	Pro-Vax, Anti-Corruption	November 13, 2020
Other: World Bank Group	Remarks by World Bank Group President David Malpass at the Gavi "One World Protected" COVAX Advance Market Commitment Investment Opportunity Launch	April 15, 2021
Other: The People's Vaccine	The Great Vaccine Robbery: Pharmaceutical corporations charge excessive prices for COVID-19 vaccines while rich countries block faster and cheaper route to global vaccination	July 29, 2021
Other: Oxfam	Vaccine Monopolies Make Cost of Vaccinating The World Against COVID At Least 5 Times More Expensive Than It Could Be	July 29, 2021
Other: World Bank Group	World Bank Support for Country Access to COVID-19 Vaccines	2021
Other: International Monetary Fund, World Bank Group, WHO, and World	Who We Are	2021

Trade Organization		
Other: U4 Anti-Corruption Resource Centre	Vaccine hesitancy, institutional mistrust and corruption in sub-Saharan Africa: everything is connected	May 20, 2021
